# Dynamic cerebral autoregulation is impaired in Veterans with Gulf War Illness: A case-control study

**DOI:** 10.1371/journal.pone.0205393

**Published:** 2018-10-15

**Authors:** Michael J. Falvo, Jacob B. Lindheimer, Jorge M. Serrador

**Affiliations:** 1 War Related Illness and Injury Study Center, Department of Veterans Affairs, East Orange, New Jersey, United States of America; 2 Department of Pharmacology, Physiology and Neuroscience, New Jersey Medical School, Rutgers, The State University of New Jersey, Newark, New Jersey, United States of America; 3 Department of Physical Medicine and Rehabilitation, New Jersey Medical School, Rutgers, The State University of New Jersey, Newark, New Jersey, United States of America; 4 William S. Middleton Memorial Veterans Hospital, Department of Veterans Affairs, Madison, Wisconsin, United States of America; 5 Department of Kinesiology, University of Wisconsin-Madison, Madison, Wisconsin, United States of America; 6 Department of Cardiovascular Electronics, National University of Ireland Galway, Galway, Connacht, IRE; Henry Ford Health System, UNITED STATES

## Abstract

Neurological dysfunction has been reported in Gulf War Illness (GWI), including abnormal cerebral blood flow (CBF) responses to physostigmine challenge. However, it is unclear whether the CBF response to normal physiological challenges and regulation is similarly dysfunctional. The goal of the present study was to evaluate the CBF velocity response to orthostatic stress (i.e., sit-to-stand maneuver) and increased fractional concentration of carbon dioxide. 23 cases of GWI (GWI+) and 9 controls (GWI) volunteered for this study. Primary variables of interest included an index of dynamic autoregulation and cerebrovascular reactivity. Dynamic autoregulation was significantly lower in GWI+ than GWI- both for autoregulatory index (2.99±1.5 vs 4.50±1.5, *p* = 0.017). In addition, we observed greater decreases in CBF velocity both at the nadir after standing (-18.5±6.0 vs -9.8±4.9%, *p* = 0.001) and during steady state standing (-5.7±7.1 vs -1.8±3.2%, *p* = 0.042). In contrast, cerebrovascular reactivity was not different between groups. In our sample of Veterans with GWI, dynamic autoregulation was impaired and consistent with greater cerebral hypoperfusion when standing. This reduced CBF may contribute to cognitive difficulties in these Veterans when upright.

## Introduction

More than 25 years after Operations Desert Storm and Shield (Gulf War), deployed Gulf War Veterans continue to report substantially poorer health relative to non-deployed Veterans of the same era, including a higher prevalence of chronic illnesses [1]. Approximately 25–32% of Gulf War Veterans are afflicted with a particular chronic illness characterized predominantly by fatigue, musculoskeletal pain, and cognitive impairment—referred to as Gulf War Illness (GWI) [2]. Although both the etiology and underlying pathophysiology in GWI remains unresolved, neurotoxicant exposure and neurological dysfunction have received the greatest attention, respectively.

Neurological dysfunction in the form of cognitive impairment is one of the hallmark symptoms of GWI, and problems with memory [3], executive function [3–5], and mood [6] have all been described in this population. Cognitive function is modulated by changes in cerebral blood flow (CBF), as evidenced by a relationship between cerebral hypoperfusion and cognitive impairment in older adults with [7, 8] and without [9] dementia. Further, reversible cognitive impairment has been described following transient occlusion of CBF in patients with cardiovascular disease [10] as well as concussion-induced reductions of CBF among collegiate athletes [11]. Therefore, cognitive symptoms in Veterans with GWI may be attributable, in part, to reductions in CBF and/or CBF dysregulation.

Haley and colleagues have studied CBF in Veterans with GWI using both single photon emission computed tomography [12], as well as magnetic resonance imaging based arterial spin labeling [13, 14]. In comparison to controls, Veterans with GWI had lower CBF in distinct regions at rest [12, 13], and subgroups of Veterans with GWI demonstrated abnormal responses to cholinergic challenge [12–14]. Impaired cholinergic control of CBF is one of several mechanisms that may affect cerebral vasodilation [15], and work from our laboratory has recently demonstrated that enhancement of cholinergic activity attenuates the decrease in CBF observed during orthostatic stress [16].

The objective of the present study was to examine the cerebrovascular response to changes in arterial blood pressure and inspired carbon dioxide (CO_2_) to determine the role of the endothelium and smooth muscle on CBF in Veterans with GWI. The response of CBF to blood pressure changes is accomplished predominately through a myogenic response (i.e., smooth muscle) of the arterioles in the cerebral vasculature, whereas the CBF response to changes in CO_2_ occurs through both endothelial and smooth muscle signals [17–19]. By indirectly assessing the smooth muscle through manipulation of arterial blood pressure (i.e., orthostatic stress) and the endothelium (i.e., manipulating inspired CO_2_), we can better understand the disruptions in CBF at the neurovascular unit. We hypothesized that Veterans with GWI would demonstrate impaired CBF in comparison to Veterans without GWI, and that this impairment would be greatest during the CO_2_ challenge given the contributions of both smooth muscle and endothelium.

## Materials and methods

### Participants

We studied 32 Gulf War-era Veterans for this study, including 23 cases of GWI (GWI+) and 9 controls (GWI-). Case status was assigned using the Kansas criteria, [20] which involves endorsement of moderate-to-severe symptoms in ≥ 3 domain areas (i.e., fatigue, pain, neurological/cognitive/mood, skin, gastrointestinal and respiratory) that began after 1990 and persisted for ≥ 1 year. Comorbid conditions (i.e., diabetes, heart disease, stroke, lupus, multiple sclerosis, cancer, etc.) were excluded per case definition. Participants provided written informed consent after receiving verbal instruction, acknowledging their understanding of the procedures and risks. All procedures were approved by the Department of Veterans Affairs New Jersey Health Care System Institutional Review Board (IRB# 01094) and conducted under the guidelines established by the Declaration of Helsinki.

### Procedures

Participants arrived at the laboratory for a single testing session having abstained from caffeine and ≥ 2 hours post-prandial. Participants were instrumented to obtain: 1) cerebral blood flow velocity (CBFV) of the middle cerebral artery via transcranial Doppler (DWL, Compumedics), 2) beat-to-beat blood pressure (MAP) via finger photoplethysmography (Finometer MIDI; Finapres), 3) end-tidal carbon dioxide (CO_2_) via capnography (Puritan-Bennett), and 4) heart rate via 3-lead electrocardiogram. Signals were sampled at 1000 Hz and digitized (PowerLab; ADInstruments). Offline analyses were performed using custom-written software (MATLAB; Mathworks).

### Sit-to-stand maneuver and cerebral autoregulation

Following a rest period (≥ 5 min), all participants performed an orthostatic challenge test designed to induce a rapid change in blood pressure as previously described [21]. Mean values of physiological signals (CBFV, MAP, end-tidal CO_2_, and heart rate) were calculated while seated (50 s epoch) and standing (mean of 5 consecutive values at nadir of blood pressure). The average of 3 maneuvers was used for analysis and the difference between seated and standing values (Δ scores) was also computed. An autoregulatory index (ARI) was computed for each sit-to-stand maneuver using a predicted curve fit model [22], and the average of the three stands was used for comparison. To assess the effect of normal fluctuations in blood pressure on CBFV, we performed transfer function analysis on 3-minute segments while seated. Three min segments were used since our lab has previously found that transfer function assessment between 3 and 5 minutes segments were similar [23]. For this analysis, beat by beat values were linearly interpolated to a 100 Hz sampling rate and then transfer function analysis was performed using the Welch method with a 45 sec Hanning window with two thirds overlap resulting in 10 windows to average. No smoothing of windows was performed. Gain, coherence and phase were calculated in three frequency bands: very low-frequency (0.03–0.07 Hz), low (0.07–0.2 Hz), and high (0.2–0.5 Hz) using custom MATLAB code [24]. Two participants with GWI and one control were excluded from transfer function analysis due to the presence of ectopic beats. Ectopic beats may affect estimation of the power spectral density [25].

### Cerebrovascular reactivity

Cerebrovascular reactivity was assessed while participants breathed at rest (normocapnia), while inspiring 8% CO_2_, 21% O_2_, balance nitrogen (hypercapnia), and during mild hyperventilation (hypocapnia) [26, 27]. Two-minute periods for hyper- and hypocapnia were selected based on prior work [26], as well as to avoid changes in middle cerebral artery diameter that occur during extended periods (≥ 4 min) [28, 29]. Change in CBFV per mmHg CO_2_ was computed via linear regression for the entire period, and during periods of hyper- and hypocapnia.

### Heart rate and blood pressure variability, and baroreflex estimate

Time- and frequency domain measures were determined as recommended by the task force report [30] in the low frequency (LF– 0.04–0.15 Hz) and high frequency (HF– 0.15–0.4 Hz) band on three minute segments while seated to assess autonomic function. In addition we used the Lomb-Scargle periodogram which does not require interpolation of the heart period signal and has been shown to be more robust with short datasets [31]. Blood pressure variability was determined from beat-by-beat blood pressure values that were re-interpolated to 4 Hz and then analyzed using the Welch method with a 50 second Hanning window and two thirds overlap, resulting in 8 windows in a 3-minute data set. Baroreflex function was estimated from transfer function gains in the LF (0.04–0.15 Hz) and HF (0.15–0.4 Hz) bands as well by the sequence method [32]. For the sequence method we examined three beat segments within the 3-minute period. Slopes were calculated on segments in which SBP values and RR interval of each consecutive beat increased or they both decreased. Each three-beat segment was plotted and a slope was calculated using least squares. All segment slopes were then averaged to determine a spontaneous baroreflex slope. In addition, number of mismatch slopes was calculated which is determined by segments in which increases in blood pressure were associated with reducing RR intervals or the reverse.

### Statistical analysis

Group means and standard deviations (SD) for arterial blood pressure (mmHg), end-tidal CO_2_ (mmHg), and CBFV (cm/s and %) were calculated for GWI+ and GWI- at rest and during orthostatic, hypo- and hyper-capnic challenges. Means (SD) were also calculated for transfer function assessment of cerebral autoregulation, heart rate and blood pressure variability, and baroreflex estimates. Data from all outcome measures were split by group and checked for normality (Kolmogorov-Smirnov test). Between-group comparisons were conducted with a series of independent samples *t*-tests for normally distributed data and Mann-Whitney U tests for non-normally distributed data (α = 0.05). Equality of variances between groups was checked prior to the analyses. Hedges’ g (*g*) effect sizes were calculated for independent-samples *t*-tests and point-biserial *r* (*r*_pb_) was calculated for Mann-Whitney U tests. Effects sizes of 0.8 [33] and 0.37 [34] were considered to be large for *g* and *r*_pb_, respectively. Pearson correlation coefficients were calculated to determine associations between CO_2_ reactivity and autoregulation. Spearman’s rho correlations were calculated to determine associations between changes in CBFV and transfer function gains.

## Results

### Participant characteristics

Demographics and medication use are reported in Table 1. Veterans with GWI+ (49.0 ± 5.9 years) were younger than Veterans without GWI (54.1 ± 6.4 years), *p* = 0.040; however, all other measures were similar between groups. Summary scores for each domain of the Kansas screening questionnaire are also reported for descriptive purposes.

**Table 1:** Clinical characteristics of cases (GWI+; n = 23) and controls (GWI-; n = 9).

	GWI+ (n = 23)	GWI- (n = 9)
**Sex, male, n (%)**	20 (86.9%)	7 (77.8%)
**Age, yrs (mean ± SD)**	49.0 ± 5.9	54.1 ± 6.4
**Height, cm (mean ± SD)**	178.5 ± 9.2	178.4 ± 5.5
**Weight, kg (mean ± SD)**	91.3 ± 15.4	107.7 ± 31.5
**Body Mass Index, kg/m**^**2**^ **(mean ± SD)**	28.6 ± 3.8	33.7 ± 8.7
**Ethnicity/Race**		
Non-Hispanic White, n (%)	12 (52.2%)	5 (55.6%)
Non-Hispanic Black, n (%)	9 (39.1%)	2 (22.2%)
Hispanic White, n (%)	1 (4.3%)	1 (11.1%)
Hispanic Black, n (%)	-	1 (11.1%)
American Indian/Alaska Native, n (%)	1 (4.3%)	-
**Kansas GWI Domain Scores**		
Fatigue	8.8 ± 2.5	-
Pain	5.7 ± 2.6	-
Neurological/Cognitive/Mood	20.1 ± 7.9	-
Skin	1.8 ± 2.0	-
Gastrointestinal	3.3 ± 3.0	-
Respiratory	2.5 ± 2.3	-
**Medications**		
Blood pressure, n (%)	3 (13.0%)	2 (22.2%)
Cholesterol, n (%)	6 (26.1%)	1 (11.1%)
Antidepressants, n (%)	5 (21.7%)	2 (22.2%)
Pain, n (%)	4 (17.4%)	0 (0.0%)
Gastrointestinal, n (%)	7 (30.4%)	1 (11.1%)

### Sit-to-stand maneuver

**Fig 1:**
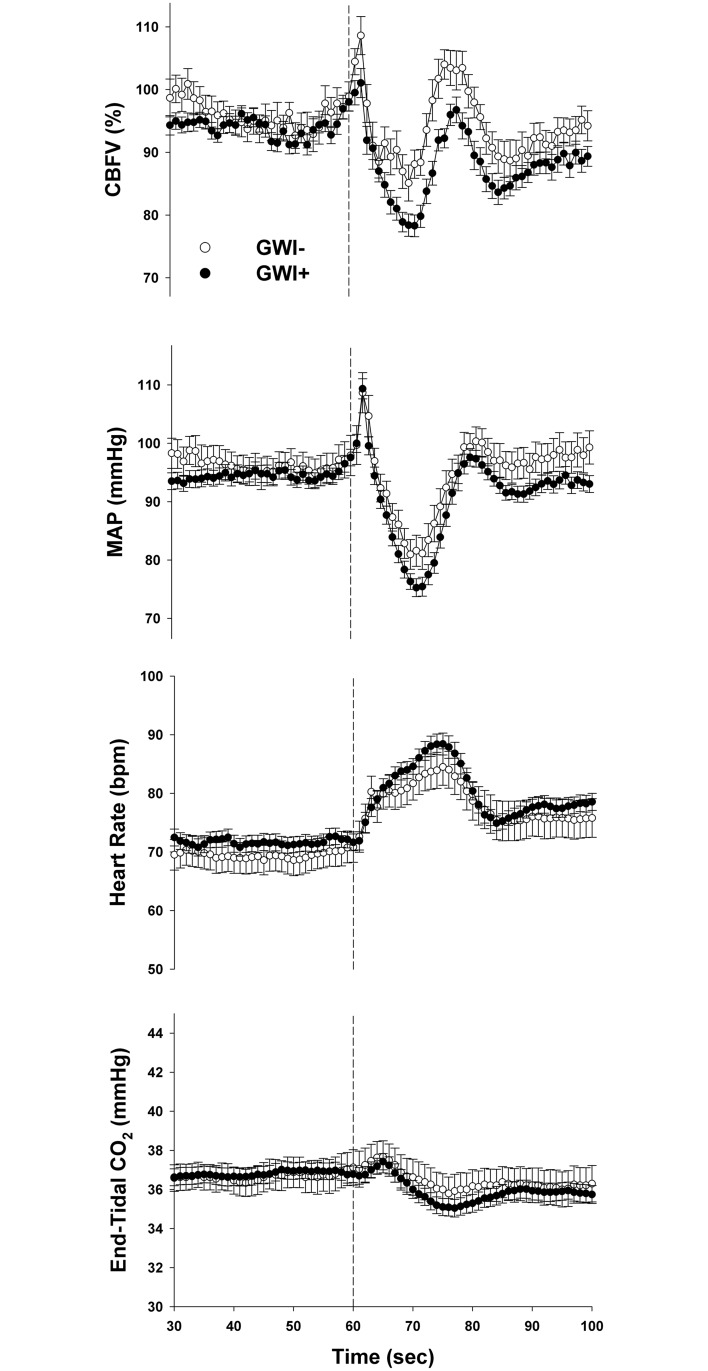
Group-averaged response for sit-to-stand maneuvers. Physiological responses are averaged across individuals and repeated sit-to-stand maneuvers. From top to bottom, cerebral blood flow velocity (CBFV) expressed as a percentage of rest, mean arterial pressure (MAP) in mmHg, heart rate in beats per minute (BPM), and end-tidal carbon dioxide (CO_2_) in mmHg averaged second-by-second. Closed and open circles represent cases with (GWI+) and controls (GWI-), respectively. Error bars are standard errors.

Group-averaged responses to the sit-to-stand maneuver are illustrated in Fig 1. Values for hemodynamic variables and their between-group comparison are reported in Table 2. CBFV obtained during seated rest (baseline) was similar between groups; however, upon standing, the drop (Δ) in CBFV from seated to standing position was significantly larger in GWI+ than GWI- despite a similar drop in blood pressure. Examining the steady state changes from seated to standing (30–55 s after stand), mean arterial pressure was lower in the GWI+ group (*p* = 0.014), although only ~3 mmHg which was likely not physiologically significant. Similarly, CBFV was lower in the GWI+ group (*p* = 0.042).

**Table 2:** Between-group comparison of autonomic responses to physiological stressor tasks.

	GWI+ Mean (SD)	GWI- Mean (SD)	p	Effect size
**Baseline Values While Sitting**				
Mean CBFV (cm/s)^B^	51.0 (15.6)	55.5 (11.9)	0.446	-0.30
Systolic CBFV (cm/s)^B^	73.7 (23.0)	82.6 (20.0)	0.318	-0.40
Diastolic CBFV (cm/s)^B^	35.9 (11.0)	38.5 (9.3)	0.542	-0.24
Mean Arterial Pressure (mmHg)^B^	94.8 (11.1)	93.0 (15.1)	0.709	0.15
Systolic Blood Pressure (mmHg)^B^	135.3 (13.6)	137.6 (17.2)	0.695	-0.16
Diastolic Blood Pressure (mmHg)^B^	76.3 (11.1)	73.0 (13.2)	0.482	0.28
Heart Rate (bpm) ^B^	71.8 (9.7)	69.4 (12.3)	0.562	0.23
End-Tidal CO2 (mmHg) ^C^	37.3 (3.4)	37.3 (3.2)	0.983	-0.004
CVR Baseline (mmHg/%) ^B^	0.99 (0.13)	0.96 (0.17)	0.638	0.21
**Nadir after Stand (5 beat average)**				
Δ MCA CBFV (%)^B^	-18.5 (6.0)	-9.8 (4.9)	**0.001**	-1.52
Δ MAP (mmHg)^C^	-19.8 (7.2)	-15.7 (4.6)	0.173	-0.24
Time to BP Nadir (s)^B^	10.2 (1.3)	10.4 (2.4)	0.808	-0.12
Time to CBFV Nadir (s)^B^	8.4 (2.2)	7.4 (2.8)	0.297	0.42
**Steady State Standing (30–55 s after stand)**				
Δ MCA CBFV (%)^B^	-5.7 (7.1)	-1.8 (3.2)	**0.042**	-0.62
Δ MAP (mmHg)^B^	-1.2 (2.3)	1.6 (3.8)	**0.014**	-1.01
Δ HR (bpm)^B^	7.2 (5.3)	6.1 (4.6)	0.593	0.21
Δ End-Tidal CO2 (mmHg)^B^	-0.98 (1.29)	-0.31 (1.0)	0.172	-0.55
Δ CVR (mmHg/%)^B^	0.051 (0.087)	0.042 (0.056)	0.776	0.11
**Dynamic Characteristics of Sit to Stand**				
Autoregulatory Index^B^	2.99 (1.49)	4.50 (1.56)	**0.017**	-1.0
VLF Gain (%/mmHg) ^C^^,^^D^	1.12 (0.40)	1.00 (0.48)	0.326	0.28
VLF Coherence^B^^,^^D^	0.48 (0.16)	0.45 (0.17)	0.627	0.18
VLF Phase (degrees)^B^^,^^D^	45.9 (31.9)	36.8 (18.5)	0.431	0.32
LF Gain (%/mmHg)^C^^,^^D^	1.48 (0.42)	1.12 (0.21)	**0.045**	0.37
LF Coherence^B^^,^^D^	0.70 (0.14)	0.50 (0.16)	**0.004**	1.37
LF Phase (degrees)^C^^,^^D^	36.9 (14.5)	41.9 (11.9)	0.304	-0.19
HF Gain (%/mmHg)^B^^,^^D^	1.73 (0.29)	1.48 (0.21)	**0.019**	0.93
HF Coherence^B^^,^^D^	0.62 (0.16)	0.68 (0.14)	0.349	-0.39
HF Phase (degrees)^C^^,^^D^	11.6 (9.3)	12.4 (19.3)	0.349	-0.18
**Cerebrovascular Reactivity Test**				
Mean CBFV Baseline (cm/s)^B^	49.5 (14.9)	56.1 (15.3)	0.276	-0.44
Mean CBFV Hypercapnia (cm/s)^B^	55.9 (17.5)	64.5 (15.4)	0.202	-0.51
Mean CBFV Hypocapnia (cm/s)^B^	38.4 (11.5)	43.3 (10.3)	0.297	-0.44
End-Tidal CO_2_ Baseline (mmHg)^B^	38.0 (3.1)	39.2 (3.2)	0.374	-0.38
End-Tidal CO_2_ Hypercapnia (mmHg)^B^	46.4 (4.7)	49.4 (5.1)	0.121	-0.62
End-Tidal CO_2_ Hypocapnia (mmHg)^B^	27.4 (3.9)	30.2 (4.3)	0.091	-0.70
Cerebrovascular Reactivity^B^ Entire range (%/mmHg)^B^	2.2 (0.5)	2.3 (0.7)	0.606	-0.18
Cerebrovascular Reactivity Hypercapnia (%/mmHg)^B^	1.6 (0.7)	1.6 (0.8)	0.944	0
Cerebrovascular Reactivity Hypocapnia (%/mmHg)^B^	2.6 (0.8)	2.7 (0.8)	0.804	-0.13

Comparison of blood pressure, end tidal carbon dioxide, cerebral blood flow velocity (CBFV), and cerebrovascular resistance (CVR) values among cases with Gulf War Illness (GWI+; n = 23) and controls (GWI-; n = 9) during a baseline seated position (25 s), during the transition from seated to standing (first 30 s after initiations of stand) and during a steady state standing period (30–55 s after initiation of stand). For transition period, change from baseline (Δ) was determined for 5 beat average at nadir of CBFV or mean arterial pressure (MAP). For standing period changes were derived from sitting steady state to standing steady state. Autoregulation Index was derived off dynamic change in blood pressure and CBFV based on best fit curve as previously described [22]. CBFV, MAP and end-tidal CO_2_ at baseline, hypercapnia, and hypocapnia are also reported, as well as the calculation of cerebrovascular reactivity. [26]

^A^ Effect sizes are reported as Hedges’ g for independent samples t-tests and point-biserial correlations for Mann-Whitney U tests

^B^ Data analyzed with independent samples t-test

^C^ Data analyzed with Mann-Whitney U test

^D^ Missing data for n = 2 GWI+ participants. Results are based on a reduced sample of n = 21 for GWI+ and n = 9 for GWI-.

### Cerebral autoregulation

**Fig 2:**
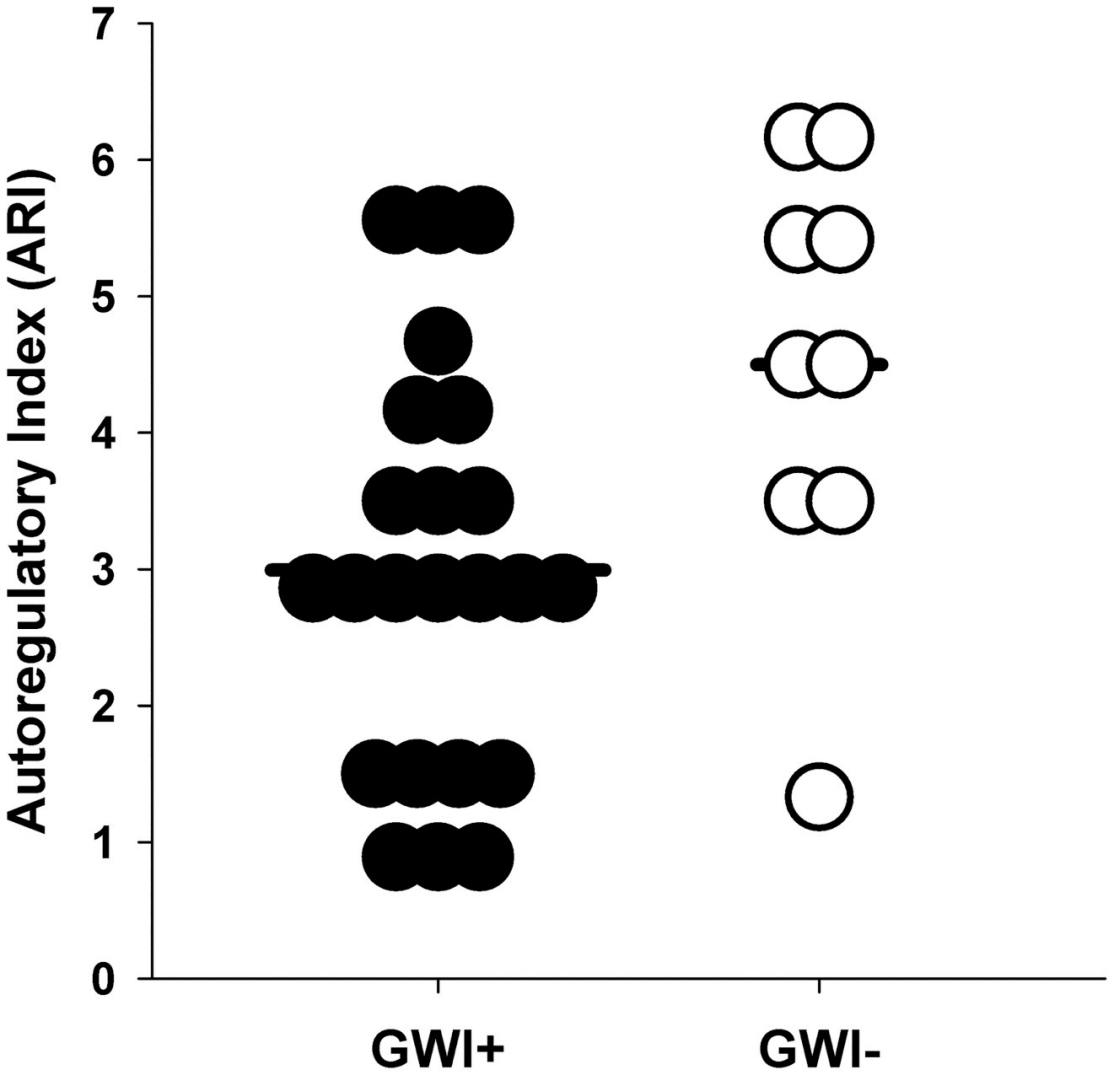
Dynamic cerebral autoregulation is impaired in Veterans with Gulf War Illness. Average autoregulatory indices were computed for three sit-to-stand maneuvers in cases with Gulf War Illness (GWI+) and controls (GWI-), respectively. Veterans with GWI+ had a significantly lower index than GWI-, consistent with impaired autoregulation.

**Fig 3:**
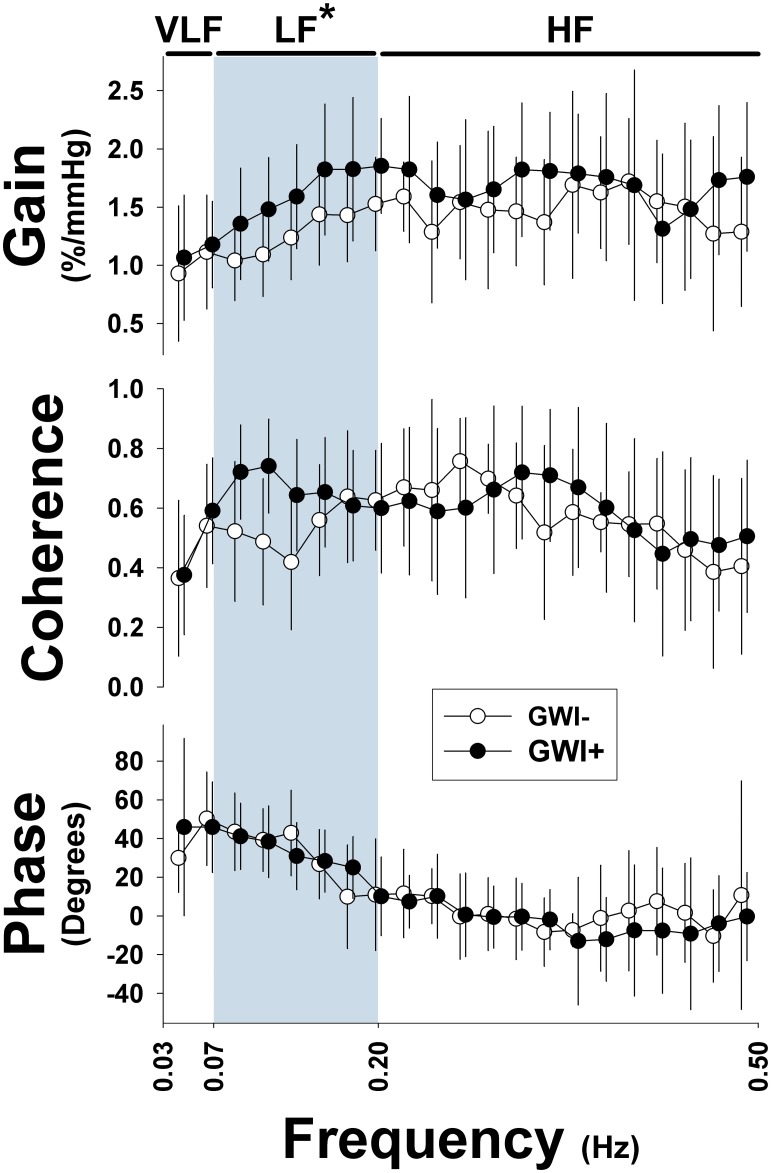
Transfer function estimates of cerebral autoregulation. From top to bottom, gain, coherence and phase between mean arterial pressure (mmHg) and cerebral flow velocity (%) over the entire frequency spectrum from the very low frequency (VLF: 0.04–0.07), low frequency (LF: 0.07–0.2 Hz) and high frequency (HF: 0.2–0.5 Hz) bands for cases with Gulf War Illness (GWI+) and controls (GWI-). Filled and open circles represent cases (GWI+) and controls (GWI-), respectively. Data are presented as mean ± SE. *, significant difference between GWI+ and GWI- for Gain only, p<0.05.

Estimated ARI was significantly lower in GWI+ than GWI- (Table 2; Fig 2). Examination of transfer function response (Fig 3) demonstrated that gain in the low, but not high frequency band, was higher in the GWI+ (Fig 3). Coherence and phase was similar in both groups at both low and high frequency bands (S2 Table). Similarly, there were no differences in gain, phase or coherence in the very low frequency band between groups.

### Cerebrovascular reactivity

Hemodynamic variables and CBFV were similar between groups at rest as well as during hyper- and hypocapnic stimulus. CO_2_ reactivity was also similar when assessed separately for hyper- and hypocapnic stimulus as well as in combination (Table 2).

### Heart rate and blood pressure variability, and baroreflex estimate

**Fig 4:**
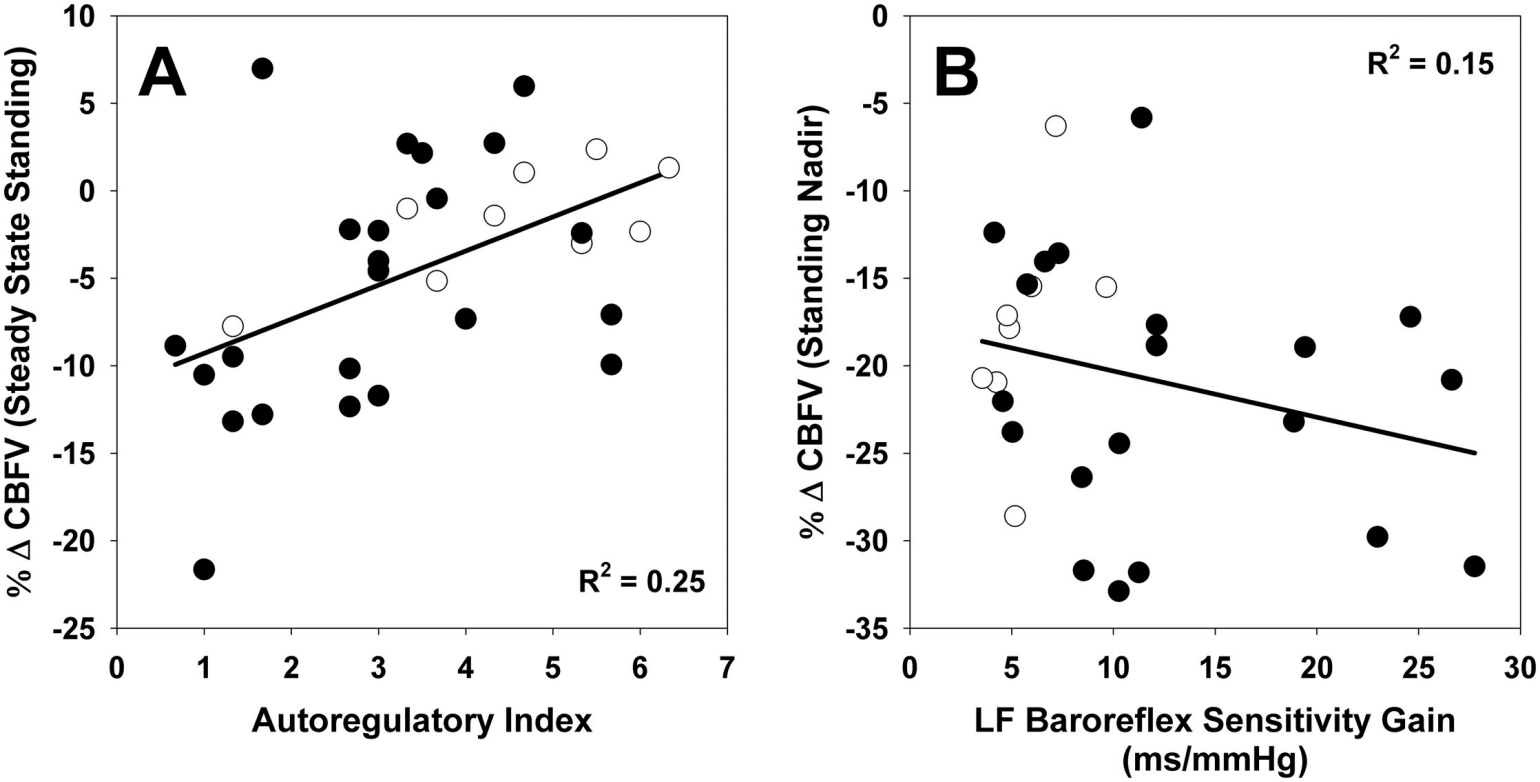
Relationship between cerebral blood flow regulation and baroreflex. Panel A: Correlation between autoregulatory index and the percent change (Δ) in cerebral blood flow velocity (CBFV) during steady state standing (30–55 sec following stand) relative to sitting ([((Standing CBFV – Sitting CBFV)/Sitting CBFV)*100]). Panel B: Correlation between low frequency (LF: 0.04–0.15 Hz) baroreflex sensitivity gain derived from transfer function estimate and the percent decrease in CBFV during nadir (5 beat average), ([((Standing CBFV @ Nadir – Sitting CBFV)/Sitting CBFV)*100]). Filled and open circles represent cases (GWI+) and controls (GWI-), respectively. Regression lines are plotted using all data, and associated R^2^ values were significant (p<0.05).

Time- and frequency-domain results for heart rate and blood pressure variability as well as baroreflex estimates are reported in the S1 and S2 Tables, respectively. Few differences were observed between-groups for heart rate and blood pressure variability (S1 Fig). In contrast, baroreflex sensitivity gains were significantly higher in the GWI+ group in the low frequency band (S2 Table and S1 Fig). We also examined how baroreflex sensitivity was related to changes in CBFV, and observed a significant correlation with the decrease in CBFV when standing (5 beat average at nadir), *R*^2^ = 0.146, *p* = 0.044(Fig 4B).

## Discussion

This is the first study to assess dynamic cerebral autoregulation in Veterans with GWI, which we found was significantly impaired relative to Veterans without GWI (Table 2; Figs 2 and 3), and the level of impairment was similar to that reported for patients with carotid artery stenosis [35] and ischemic stroke [36]. Contrary to our hypothesis, CO_2_ reactivity was similar between Veterans with and without GWI. Given that CBF dysregulation in the present study was observed during active transition to an upright posture, we suggest that disturbances among other systems (e.g., autonomic, vestibular, bioenergetics) may contribute to these results.

Prior studies have assessed the response to orthostatic stress in GWI using tilt-table testing with [37, 38] and without isoproterenol [39] as well as a clinical wall lean test [40] with mixed results in terms of hypotension but similar reports of orthostatic symptoms. However, none of these prior studies assessed CBF which is noteworthy as orthostatic symptoms in the absence of hypotension may reflect an underlying orthostatic cerebral hypoperfusion [41]. For example, Novak recently described a tilt-induced drop in CBF of approximately -24% in 102 patients with idiopathic orthostatic symptoms in comparison to -4.2% decrease in controls, in the absence of orthostatic hypotension [41]. Direct comparison of our results is not possible as we did not employ similar tilt-table protocols. Rather, the present study utilized the sit-to-stand maneuver, which unlike passive tilt that may take up to 12 s to transition from supine to upright, takes < 5 s to transition from sitting to standing thereby affording assessment of dynamic cerebral autoregulation [21]. In this work, Veterans with GWI displayed a marked reduction in CBFV in comparison to controls that is not fully explained by concomitant decreases in blood pressure and end-tidal CO2 (Fig 1). Moreover, we also found that impaired autoregulation (i.e., lower ARI values) was associated with a greater reduction in steady state CBFV when standing (Fig 4A).

Further support for impaired dynamic cerebral autoregulation in this group is derived from the transfer function analysis of steady state sitting values. Veterans with GWI had higher transfer function gains in both the low and high frequency bands, suggesting they were less effective at minimizing the impacts of changes in blood pressure on CBF (Fig 3). Similarly, the greater coherence in the low frequency band also suggests that CBF changes were related to blood pressure changes. However, phase, another indicator of autoregulation was unchanged. While there is no previous data that has examined transfer function gains in GWI, we have previously published that a cohort of 193 males with a mean age of 78.3 years had mean gains of 1.34 [42], less than the mean seen in these Veterans with GWI. Thus, despite the Veterans with GWI having a mean age of 49 years, their CBF regulation was worse than a cohort of elderly.

To assess the vasodilatory capacity of the cerebral vasculature, we examined changes in CBF velocity of the middle cerebral artery in response to changes in end-tidal CO_2_ [43]. We hypothesized that Veterans with GWI would demonstrate attenuated CO_2_ reactivity relative to controls, suggesting a decreased capacity to dilate the cerebral vessels. However, we observed similar CO_2_ reactivity between-groups. This suggests that factors other than CO_2_ reactivity contribute to the autoregulatory response, most notably cholinergic activation of the cerebral vasculature [44]. In support, we recently demonstrated in a randomized double-blind study that physostigmine infusion enhanced CBF velocity despite the presence of hypocapnia [16]. An active cholinergic vasodilatory reflex has been proposed to counter sympathetic vasoconstriction in the cerebral circulation [15], and this reflexive response appears disturbed in GWI [13, 14] which may be consistent with broader autonomic dysfunction in this population [39].

To measure autonomic function in this group, we examined heart rate and blood pressure variability. We found no evidence of differences in indicators of cardiac parasympathetic control unlike previous works that have demonstrated reduction in high frequency HRV [45–47]. This might be due to the fact that previous work examined 24 hour recordings, compared to our short 3-min periods. However, Haley et al. [47] also found no difference during the day when Veterans were awake, which supports our daytime recordings. To examine indicators of sympathetic control of the heart and periphery, we examined LF HRV and blood pressure variability. We found that cardiac sympathetic control was elevated (LF HRV power) but there was no difference in peripheral sympathetic activity (LF blood pressure power). Our results contrast Stein et al. [45] who found reduced LF HRV in 24 hour recordings. Our peripheral results are similar to those of Haley et al. [46] who found no significant difference in sympathetic nerve activity between groups. The difference in our HRV findings may be due to the analysis method. When using traditional HRV FFT methods we did not find a significant increase in LF HRV. However, using a novel analysis method that has been shown to be more sensitive since it does not require interpolation of the RR interval sequence [31], we did detect a difference. In addition, both LF HRV and BP variability are non-invasive indicators of sympathetic activity. However, they are not direct measures and have many other inputs which affect the response. Thus, these data are only indicative of changes in autonomic function. Regardless, our data as well as previous data suggests that autonomic function is affected in GWI. However, none of our measures examined autonomic control of the cerebrovasculature.

Another novel finding in this work is the effect of GWI on baroreflex sensitivity. To our surprise we found that baroreflex sensitivity assessed by transfer function was significantly greater in Veterans with GWI. Despite having improved baroreflex, steady state standing blood pressure was significantly lower, although only slightly (~3 mmHg), in the veterans with GWI despite improved baroreflex sensitivity. The reason for this remains unclear and further work on central cardiac function and total peripheral resistance changes when upright is necessary. It is also unclear why baroreflex would be improved in veterans with GWI. One theoretical possibility is that to help maintain CBF when cerebral autoregulation is impaired, tighter regulation of blood pressure is necessary to ensure adequate perfusion of the brain. Several previous studies have found an inverse relationship between baroreflex and cerebral autoregulation in healthy young individuals [48–50]. Based on our findings this same relationship is true for Veterans with GWI since those with the lowest ARI values had the highest baroreflex transfer function gains (Fig 4B).

As impairments in dynamic autoregulation were observed upon standing, our findings may also suggest a role for vestibular input to the cerebrovasculature. In support of this possibility, Haley and colleagues [51] found that Veterans with GWI classified as having vestibular ataxia and vertigo attacks not only exhibit the greatest functional impairment [52], but also demonstrate the largest reduction in CBF at baseline and largest increase in CBF secondary to physostigmine challenge [12]. Though the relationship between the vestibular system and CBF remains unclear in humans, we have previously found that stimulation of the vestibular system results in modulation of CBF and that this regulation was independent of blood pressure and end-tidal CO_2_ [53]. Prior studies in GWI have also noted a role for vestibular dysfunction in GWI symptomatology [54], that is independent of stress and/or anxiety [55], and objectively worse in Veterans with GWI in comparison to controls [55]. Moreover, episodes of syncope [56] and dizziness [57, 58], reported in GWI could be interpreted by either impaired CBF regulation and/or vestibular dysfunction. No published work to date has comprehensively examined vestibular function in GWI or whether vestibular dysfunction affects cerebral autoregulation in GWI.

An important aspect of this work is that measures of CBF were performed upright. Our recent work demonstrating that cholinergic enhancement was only effective in improving CBF in healthy subjects when upright highlights the importance of orthostatic stress in considering CBF response [16]. Further support for the importance of orthostatic stress comes from work in chronic fatigue syndrome in which patients not only demonstrated greater drops in CBFV during head up tilt but also impaired cognitive performance when upright but not supine [59]. Since cognitive complaints in Veterans with GWI are made when they are upright, we must consider the importance of studying the upright posture. If cholinergic inputs are involved in cerebral vasodilation when upright and cholinergic function is impaired in GWI, enhancing cholinergic function could provide a target for treatment. Note that in our previous work the improvement in CBF did not occur with neostigmine but only with physostigmine, a cholinesterase inhibitor that can cross the blood brain barrier. Future work is needed to examine if cerebrovascular cholinergic mechanisms are impaired in Veterans with GWI when upright.

Notwithstanding the limitations of a cross-sectional study, there are other factors that may impact the interpretation of our findings. GWI is a heterogeneous disorder; therefore, our relatively small sample size, poor representation of female veterans, and unequal group sizes are limitations. Human subject research in GWI is challenging as common conditions of advanced age are exclusions for GWI case status. Despite this, we observed a large effect size for a primary variable of interest, ARI. However, we were likely sufficiently underpowered to detect a meaningful change in certain secondary variables. Another possible consideration for differences between groups is due to physical activity. GWI is a fatiguing illness that makes most unable to exercise and thus results in the veterans becoming extremely sedentary. Thus, impaired autoregulation in the veterans with GWI could be the result of deconditioning. While we did not have measures of physical activity or fitness in the two groups previous work examining fit vs sedentary elderly found no difference in cerebral autoregulation [60].

### Conclusions

In summary, Veterans with GWI demonstrate dysregulation of CBF during transition from a seated to standing position and when standing. Reasons for the disrupted regulation of CBF are likely multifactorial and may be manifested by disturbances in other physiological systems (i.e., autonomic and vestibular) and bioenergetics (i.e., mitochondrial dysfunction). Future studies are needed to expand on this work, including association of impaired cerebral blood regulation when upright and impaired cognitive function as well as in relation to symptoms at rest and during symptom exacerbation (i.e., post-exertion malaise).

## Supporting information

S1 Table Heart rate & blood pressure variability. Comparison of measures of heart rate and blood pressure variability among Veterans who screened positive for Gulf War Illness (n = 23) and healthy controls (n = 9) during a 2–3 min steady state period while seated. For time domain measures, mean values of RR Interval (Mean RR) as well as standard deviation (SDNN) and root mean square of successive differences between RR intervals (rMSSD) were obtained. Heart rate variability was derived using the Power Spectrum (Welch) and Power Spectrum (Lomb-Scargle) periodgrams in the low frequency (LF: 0.04–0.15 Hz) and the high frequency (HF: 0.14–0.4 Hz) bands. Values were calculated for power in absolute units as well as % of total power. Ratios of low frequency to high frequency power (LF/HF) was derived. Variability of systolic blood pressure (SBP) was also calculated in the LF and HF bands. ^A^ Effect sizes are reported as Hedges’ g for independent samples t-tests and point-biserial correlations for Mann-Whitney U tests; ^B^ Data analyzed with independent samples t-test; ^C^ Data analyzed with Mann-Whitney U test.
(DOCX)

S2 Table Baroreflex estimate. Comparison of measures of baroreflex sensitivity (BRS) among Veterans who screened positive for Gulf War Illness (n = 23) and healthy controls (n = 9) during a 2–3 min steady state period while seated. Transfer function estimates of gain, coherence and phase were obtained in the low frequency (LF: 0.04–0.15 Hz) and the high frequency (HF: 0.14–0.4 Hz) bands. Spontaneous baroreflex was also estimated from 3 beat segments of systolic blood pressure and RR interval to obtain a mean slope as well as the number of mismatched segments. ^A^ Effect sizes are reported as Hedges’ g for independent samples t-tests and point-biserial correlations for Mann-Whitney U tests; ^B^ Data analyzed with independent samples t-test; ^C^ Data analyzed with Mann-Whitney U test.
(DOCX)

S1 Fig Heart rate variability and Baroreflex. Upper left corner displays low frequency heart rate variability (LF HRV) in the low frequency band (0.04–0.15 Hz) band for Veterans with GWI (GWI+) and controls (GWI-). Lower two panels represent baroreflex sensitivity (BRS) gain derived from transfer function estimates in the low frequency (0.04–0.15 Hz), left panel, and high frequency (0.15–0.4 Hz), right panel, bands. Filled dots are individual values of Veterans with GWI, while open dots are controls. Solid lines represent mean values. *, significant difference between GWI+ and GWI- in that frequency band, p<0.05).
(DOCX)
