# Supplementary material for: Dynamic cerebral autoregulation is impaired in Veterans with Gulf War Illness: A case-control study
Source: PLoS One. 2018 Oct 15;13(10):e0205393. doi: 10.1371/journal.pone.0205393 (PMC6188758; doi:10.1371/journal.pone.0205393)
Supplement: S1 Table — Comparison of measures of heart rate and blood pressure variability among Veterans who screened positive for Gulf War Illness (n = 23) and healthy controls (n = 9) during a 2–3 min steady state period while seated. For time domain measures, mean values of RR Interval (Mean RR) as well as standard deviation (SDNN) and root mean square of successive differences between RR intervals (rMSSD) were obtained. Heart rate variability was derived using the Power Spectrum (Welch) and Power Spectrum (Lomb-Scargle) periodgrams in the low frequency (LF: 0.04–0.15 Hz) and the high frequency (HF: 0.14–0.4 Hz) bands. Values were calculated for power in absolute units as well as % of total power. Ratios of low frequency to high frequency power (LF/HF) was derived. Variability of systolic blood pressure (SBP) was also calculated in the LF and HF bands. A Effect sizes are reported as Hedges’ g for independent samples t-tests and point-biserial correlations for Mann-Whitney U tests; B Data analyzed with independent samples t-test; C Data analyzed with Mann-Whitney U test. (DOCX) [file pone.0205393.s001.docx]

**S1 Table. – Heart Rate & Blood Pressure Variability**

|  | **Gulf War Illness**  **Mean (SD)** | **Control**  **Mean (SD)** | **p** | **Effect size^A^** |
| --- | --- | --- | --- | --- |
| **Time Domain Measures** |  |  |  |  |
| Mean RR (ms)^B^ | 836 (94.8) | 884.9 (150.6) | 0.302 | -0.44 |
| SDNN (ms)^C^ | 34.8 (15.5) | 33.2 (11.5) | 0.943 | -0.02 |
| rMSSD (ms^2^)^C^ | 21.7 (15.7) | 22.2 (12.2) | 0.684 | -0.08 |
| **FFT Measures (Welch)** |  |  |  |  |
| LF (ms^2^)^C^ | 332.6 (320.7) | 241.1 (212.7) | 0.457 | 0.15 |
| LF (%)^B^ | 67.1 (16) | 61.4 (19.7) | 0.423 | 0.33 |
| HF(ms^2^)^C^ | 172.3 (219) | 145.8 (114.1) | 0.684 | -0.08 |
| HF (%)^B^ | 32.9 (16) | 38.6 (19.7) | 0.423 | -0.33 |
| LF/HF^C^ | 3.2 (3.0) | 2.2 (1.6) | 0.582 | 0.11 |
| **FFT Measures (Lomb-Scargle)** |  |  |  |  |
| LF (ms^2^)^B^ | 1.6 (0.5) | 1.1 (0.5) | **0.033** | 1.0 |
| LF (%)^B^ | 81.8 (10) | 77.1 (15.5) | 0.346 | 0.40 |
| HF(ms^2^)^C^ | 0.33 (0.17) | 0.31 (0.28) | 0.401 | 0.16 |
| HF (%)^B^ | 18.2 (10) | 22.9 (15.5) | 0.346 | -0.40 |
| LF/HF^C^ | 7.1 (6.6) | 5.2 (3.7) | 0.549 | 0.12 |
| **Blood Pressure Variability** |  |  |  |  |
| SBP LF (mmHg^2^)^C^ | 70.1 (38.1) | 98.3 (80.7) | 0.649 | -0.09 |
| SBP HF(mmHg ^2^)^C^ | 10.4 (10.8) | 14.7 (13.4) | 0.457 | -0.15 |

Comparison of measures of heart rate and blood pressure variability among Veterans who screened positive for Gulf War Illness (n=20) and healthy controls (n=8) during a 2-3 min steady state period while seated. For time domain measures, mean values of RR Interval (Mean RR) as well as standard deviation (SDNN) and root mean square of successive differences between RR intervals (rMSSD) were obtained. Heart rate variability was derived using the Power Spectrum (Welch) and Power Spectrum (Lomb-Scargle) periodgrams in the low frequency (LF: 0.04-0.15 Hz) and the high frequency (HF: 0.14-0.4 Hz) bands. Values were calculated for power in absolute units as well as % of total power. Ratios of low frequency to high frequency power (LF/HF) was derived. Variability of systolic blood pressure (SBP) was also calculated in the LF and HF bands.

^A^ Effect sizes are reported as Hedges’ g for independent samples t-tests and point-biserial correlations for Mann-Whitney U tests

^B^ Data analyzed with independent samples t-test

^C^ Data analyzed with Mann-Whitney U test
