# Supplementary material for: Dynamic cerebral autoregulation is impaired in Veterans with Gulf War Illness: A case-control study
Source: PLoS One. 2018 Oct 15;13(10):e0205393. doi: 10.1371/journal.pone.0205393 (PMC6188758; doi:10.1371/journal.pone.0205393)
Supplement: S2 Table — Comparison of measures of baroreflex sensitivity (BRS) among Veterans who screened positive for Gulf War Illness (n = 23) and healthy controls (n = 9) during a 2–3 min steady state period while seated. Transfer function estimates of gain, coherence and phase were obtained in the low frequency (LF: 0.04–0.15 Hz) and the high frequency (HF: 0.14–0.4 Hz) bands. Spontaneous baroreflex was also estimated from 3 beat segments of systolic blood pressure and RR interval to obtain a mean slope as well as the number of mismatched segments. A Effect sizes are reported as Hedges’ g for independent samples t-tests and point-biserial correlations for Mann-Whitney U tests; B Data analyzed with independent samples t-test; C Data analyzed with Mann-Whitney U test. (DOCX) [file pone.0205393.s002.docx]

**S2 Table. Baroreflex Estimate**

|  | **Gulf War Illness**  **Mean (SD)** | **Control**  **Mean (SD)** | **p** | **Effect size^A^** |
| --- | --- | --- | --- | --- |
| **Transfer Function** |  |  |  |  |
| BRS Gain LF (ms/mmHg)^C^ | 12.9 (7.7) | 5.7 (1.9) | **0.004** | 0.53 |
| BRS Gain HF (ms/mmHg)^C^ | 14.3 (10.6) | 7.7 (4.2) | 0.200 | 0.24 |
| BRS Coherence LF^B^ | 0.73 (0.10) | 0.76 (0.05) | 0.575 | -0.34 |
| BRS Coherence HF^B^ | 0.72 (0.08) | 0.69 (0.06) | 0.301 | 0.40 |
| BRS Phase LF (deg)^B^ | -50.6 (27.9) | -40.7 (21.6) | 0.376 | -0.38 |
| BRS Phase HF (deg)^B^ | 30.2 (39.3) | 33.8 (44.5) | 0.830 | -0.09 |
| **Spontaneous Baroreflex** |  |  |  |  |
| Slope (ms/mmHg)^C^ | 17.3 (12.4) | 11.1 (5.8) | 0.182 | 0.26 |
| Mismatch (N)^C^ | 22.3 (13.0) | 19.0 (11.8) | 0.582 | 0.10 |

Comparison of measures of baroreflex sensitivity (BRS) among Veterans who screened positive for Gulf War Illness (n=21) and healthy controls (n=8) during a 2-3 min steady state period while seated. Transfer function estimates of gain, coherence and phase were obtained in the low frequency (LF: 0.04-0.15 Hz) and the high frequency (HF: 0.14-0.4 Hz) bands. Spontaneous baroreflex was also estimated from 3 beat segments of systolic blood pressure and RR interval to obtain a mean slope as well as the number of mismatched segments.

^A^ Effect sizes are reported as Hedges’ g for independent samples t-tests and point-biserial correlations for Mann-Whitney U tests

^B^ Data analyzed with independent samples t-test

^C^ Data analyzed with Mann-Whitney U test
