# Supplementary material for: Dynamic cerebral autoregulation is impaired in Veterans with Gulf War Illness: A case-control study
Source: PLoS One. 2018 Oct 15;13(10):e0205393. doi: 10.1371/journal.pone.0205393 (PMC6188758; doi:10.1371/journal.pone.0205393)
Supplement: S1 Fig — Upper left corner displays low frequency heart rate variability (LF HRV) in the low frequency band (0.04–0.15 Hz) band for Veterans with GWI (GWI+) and controls (GWI-). Lower two panels represent baroreflex sensitivity (BRS) gain derived from transfer function estimates in the low frequency (0.04–0.15 Hz), left panel, and high frequency (0.15–0.4 Hz), right panel, bands. Filled dots are individual values of Veterans with GWI, while open dots are controls. Solid lines represent mean values. *, significant difference between GWI+ and GWI- in that frequency band, p<0.05). (DOCX) [file pone.0205393.s003.docx]

**S1 Fig. Heart Rate Variability and Baroreflex.**

Upper left corner displays low frequency heart rate variability (LF HRV) in the low frequency band (0.04-0.15 Hz) band for Veterans with GWI (GWI+) and controls (GWI-). Lower two panels represent baroreflex sensitivity (BRS) gain derived from transfer function estimates in the low frequency (0.04-0.15 Hz), left panel, and high frequency (0.15-0.4 Hz), right panel, bands. Filled dots are individual values of Veterans with GWI, while open dots are controls. Solid lines represent mean values. *, significant difference between GWI+ and GWI- in that frequency band, p<0.05)
